# Life-Threatening Sublingual Hematoma in the Setting of Anticoagulation and Neck Trauma

**DOI:** 10.7759/cureus.24974

**Published:** 2022-05-13

**Authors:** Derrick Huang, Rohan Wanchu, Joshua Walker, Latha Ganti

**Affiliations:** 1 Emergency Medicine, HCA Florida Ocala Hospital, Ocala, USA; 2 Emergency Medicine, University of Central Florida College of Medicine, Orlando, USA; 3 Emergency Medicine, Envision Physician Services, Plantation, USA

**Keywords:** neck trauma, life-threatening bleeding, acute airway obstruction, airway bleeding, macroglossia

## Abstract

Sublingual hematoma is a rare and life-threatening emergency department presentation. The rich vascular supply of the tongue results in a predisposition for rapid hemorrhage secondary to lingual trauma and developing lethal upper airway obstruction. In the setting of a patient with neck trauma, assessment of risk factors, such as the use of anticoagulation, and clinical signs of vascular injury are essential for rapid diagnosis and mobilization of resources for airway protection.

## Introduction

Enlargement of the tongue due to sublingual hematoma resulting from neck trauma can lead to life-threatening upper airway emergencies [1-2]. The rich vascularity of the tongue leaves it susceptible to rapid hemorrhage after trauma or vascular insult [2-3]. This outflow of blood can result in massive tongue enlargement with cephalad and posterior displacement and subsequent lethal upper airway obstruction [2]. Primary etiologies of lingual hematoma include oropharyngeal trauma, such as motor vehicle accidents, seizures, and traumatic intubations, as well as spontaneous causes, such as hypertension and vascular malformation in the lingual arterial system. These etiologies are often mediated by the use of anticoagulation therapy and coagulopathic states, such as renal and liver failure [1-3].

In the emergency department, the primary focus in the setting of neck trauma is the immediate assessment of the airway and breathing for rapid recognition of impending airway compromise and life-threatening vascular injury. For example, patients are risk-stratified by violation of the platysma, presence of soft signs, such as hemoptysis or hematemesis, dysphonia, and presence of hard signs, such as expanding hematoma, pulsatile bleeding, decreased or absent pulses, and vascular thrills, which may indicate emergent consultation and operative management [4]. Here, we present a case of a massive lingual hematoma secondary to penetrating neck trauma in a case complicated by anticoagulation use.

## Case presentation

A 63-year-old man with a past medical history of aortic valve replacement on apixaban presented to the emergency department with a gunshot wound to the face. The patient was cleaning his handgun when the weapon was accidentally fired under his chin. Initial vital signs included pulse oximetry of 99% on room air, a blood pressure of 166/101 mmHg, and a heart rate of 85 beats per minute. The patient was speaking clearly with bilateral breath sounds and neurovascularly intact extremities. He had an approximately 0.5 cm penetrating injury to the submental space with a likely platysma violation. The injury was without external bleeding, hematoma, or thrills. On oropharyngeal examination, there was no lingual displacement and the patient was able to speak without a change in voice. With a cervical collar in place, the patient was in a supine position with no respiratory distress. The patient was then taken to the CT scan for trauma imaging.

**Figure 1:**
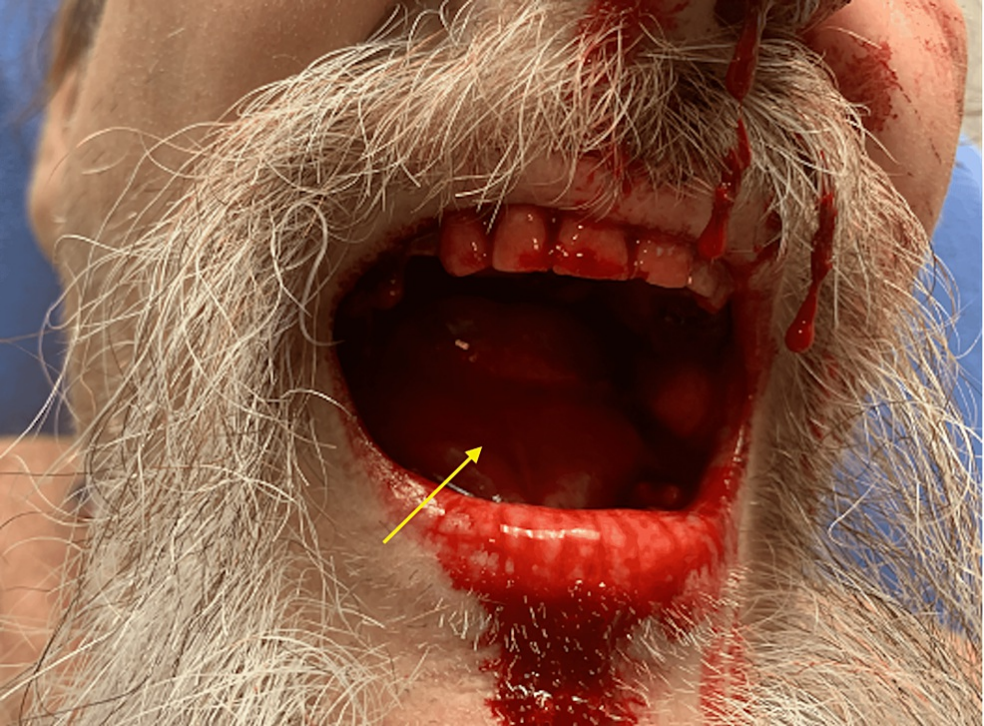
Sublingual hematoma (yellow arrow)

Upon the initial return of the patient to the ED, there were no changes on examination. Approximately 30 minutes later, the patient was found to be leaning forward with worsening respiratory distress as a result of a rapidly developing sublingual hematoma (Figure 1).

**Figure 2:**
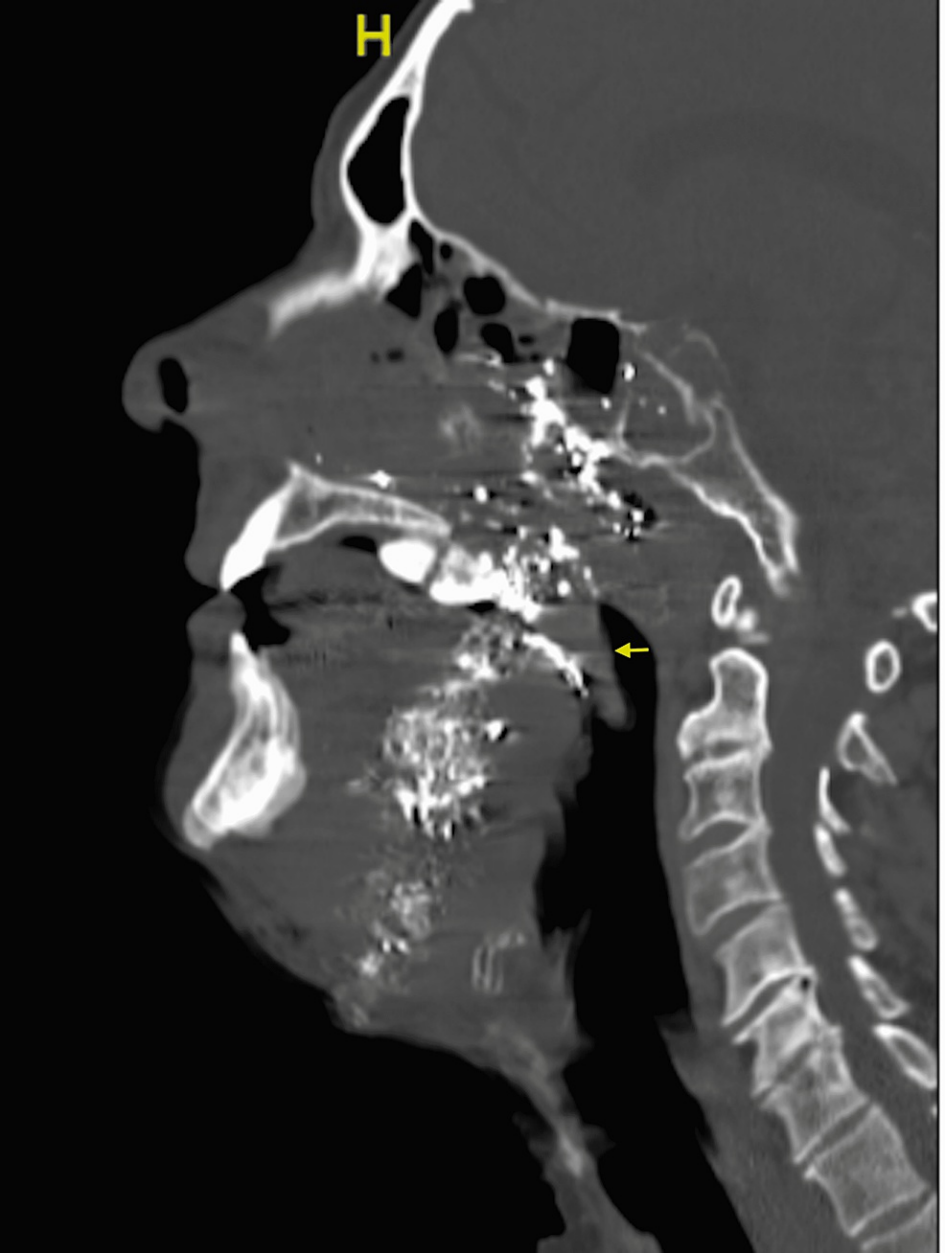
CT face without intravenous contrast with a sagittal view. Hard palate fracture (yellow arrow) and multiple bullet fragments at the sphenoid sinuses, ethmoid air cells, nasal cavity, posterior nasopharynx mucosa, and hard palate.

The patient was taken to the ICU where he was given tranexamic acid (TXA) as well as prothrombin complex concentrate (PCC) to reverse apixaban anticoagulation. Noncontrast CT of the face was remarkable for a hard palate fracture (Figure 2).

CT brain and angiography of the head and neck were without acute injury. Intravenous ampicillin with sulbactam was administered. The patient was safely extubated the next day and initiated on a course of dexamethasone for residual tongue swelling. Under consultation with facial trauma and otorhinolaryngology, the patient was recommended nonoperative management and outpatient prosthodontist follow-up for obturator placement for recurrent nasal reflux.

## Discussion

We report a case of a sublingual hematoma secondary to penetrating submental neck trauma that ultimately required intubation. Our case was complicated by a penetrating neck injury under the mandible with involvement of the tongue in the setting of a patient on anticoagulation for his aortic valve replacement. Given the rich vascularity of the tongue, lingual trauma in the setting of anticoagulation likely contributed to the delayed, sudden, and rapid sublingual hematoma formation despite our patient’s initial presentation without signs of airway compromise or sublingual hematoma [1,2]. Although our patient did not display hard or soft signs of a neck injury on arrival, these signs may be less reliable in cases of penetrating tongue injury and anticoagulation use. Clinicians should closely monitor patients with lingual trauma and have a low threshold for early intubation.

Assessment of possible etiologies and risk factors for the development of sublingual hematoma is crucial for rapid diagnosis and prompt airway protection. Acute tongue enlargement has various etiologies predominately ranging from hemorrhage secondary to direct trauma, vascular anomalies, coagulation derangements, edema, infarction, and infection [2,5]. As in our case, these factors can be synergetic in promoting hematoma formation. For example, lingual trauma in the setting of coagulation derangement, including thrombocytopenia, chronic renal failure, and anticoagulation, may be associated with profuse bleeding [1,2]. Importantly, CT imaging, including angiography, is essential in assessing the severity of sublingual hematomas in order to guide further management and diagnosing concomitant vascular, intracranial, skeletal, and further airway injury in the setting of oropharyngeal trauma [4,6].

Airway management is the foremost concern of the emergency provider and is essential before definitive treatment of sublingual hematomas. The anatomic position of the tongue predisposes a posterior-cephalad displacement upon sublingual hematoma formation, which may result in lethal upper airway obstruction [1,2]. In non-emergent cases, flexible fiberoptic laryngoscopy has been utilized to assess the degree of obstruction, which may prevent unnecessary intubations [3]. In the setting of lingual trauma, close monitoring for developing signs of respiratory distress and upper airway obstruction, such as swelling, bleeding, dysphonia, drooling, dyspnea, and stridor, is vital for prompt airway protection [2-3]. Due to the displacement of the tongue, oral endotracheal intubation may be difficult to perform, and the patient may not be amenable to bag valve mask ventilation. This may necessitate awake fiberoptic intubation via the nasal route under local anesthetic and sedation [2,7]. Additionally, a surgical airway, including an emergency cricothyrotomy or a tracheostomy in the operating room, might be required [7]. In our case, the patient was taken to the operating room and successfully intubated via oral endotracheal intubation with preparation for as-needed emergency cricothyroidotomy at the bedside [7]. Once the airway is secure, treatment may focus on the reversal of anticoagulation, steroids for lingual swelling, and antibiotics [3,8]. Other options are dependent on hematoma size and active expansion and include local control to interventional radiology embolization or surgical ligation of the lingual arteries [3]. 

## Conclusions

The rich vascularity of the tongue results in a predisposition for rapid hemorrhage and the potential development of lethal upper airway obstruction. In the setting of oropharyngeal trauma, assessment for respiratory distress, signs of neck trauma, and risk factors such as anticoagulation use, as well as imaging for concomitant injuries are essential. Lingual trauma complicated by anticoagulation use may lead to unpredictable and rapid hematoma formation with airway compromise, necessitating close airway monitoring and a low threshold for early securement of the airway. 
